# An open-access database of nature-based carbon offset project boundaries

**DOI:** 10.1038/s41597-025-04868-2

**Published:** 2025-04-05

**Authors:** Akshata Karnik, John B. Kilbride, Tristan R. H. Goodbody, Rachael Ross, Elias Ayrey

**Affiliations:** 1 Renoster Systems Inc., 21750 Hardy Oak Blvd Ste 104, PMB 37519, San Antonio, TX 78258-4946 United States of America; 2 Carbon Direct, Vancouver, British Colombia, Canada; 3 Carbon Direct, New York, New York, United States of America

**Keywords:** Forestry, Environmental impact

## Abstract

Nature-based climate solutions (NBS) have become an important component of strategies aiming to reduce atmospheric CO_2_ and mitigate climate change impacts. Carbon offsets have emerged as one of the most widely implemented NBS strategies, however, these projects have also been criticized for exaggerating offsets. Verifying the efficacy of NBS-derived carbon offset is complicated by a lack of readily available geospatial boundary data. Herein, we detail methods and present a database of nature-based offset project boundaries. This database provides the locations of 575 NBS projects distributed across 55 countries. Geospatial boundaries were aggregated using a combination of scraping data from carbon project registries (*n* = 433, 75.3%) as well as manual georeferencing and digitization (*n* = 127, 22.1%). Database entries include three varieties of carbon projects: avoided deforestation, afforestation, reforestation and re-vegetation, and improved forest management. An accuracy assessment of the georeferencing and digitizing process indicated a high degree of accuracy (intersection over union score of 0.98 ± 0.015).

## Background & Summary

Nature-based climate solutions (NBS)^1^ represent one of the most cost-efficient methods for reducing atmospheric CO_2_, performing ecosystem restoration, and achieving sustainable development goals^2,3^. Through improved management and restoration of ecosystems, atmospheric carbon can be sequestered into terrestrial carbon pools while simultaneously protecting ecosystem services and preventing the loss of biodiversity^4,5^. NBS could achieve 30% of the CO2 reductions needed to keep global warming under 2°C by 2030^6–8^. Carbon offsets, also reffered to as credits, are a transferable commodity that represents the removal of a tonne of atmospheric CO2. These credits have emerged as a key mechanism for directing public and private financial resources towards climate change mitigation^1^. The market for traded carbon credits is estimated to have grown from $295.7 million in 2018 to over $2 billion in 2021^9^.

While growth in the carbon market is promising, concerns over issuance of credits (i.e., verifiable reductions of one tonne of atmospheric carbon dioxide which can be traded/purchased to offset producer emissions), human rights violations, and accounting errors have been raised, challenging the efficacy of nature-based carbon projects^1,10–14^. NBS-derived carbon credits are most commonly issued based on a baseline emissions scenario representing what could have occurred in the absence of the projects proposed activities (e.g. avoiding planned/unplanned deforestation, afforestation)^9^. This system incentivizes project developers to maximize the number of credits issued by constructing pessimistic baselines. Problematically, project developers are given considerable latitude in the choice of forecasting algorithms, which has substantial impact in the final number of credits issued^9,15,16^. Recently, NBS projects developed under programs and frameworks such as the Reducing Emissions from Deforestation and forest Degradation (REDD+), have been criticized and accused of issuing millions of credits with no genuine emission reductions^1,12,13^.

Analyzing and validating project emission reduction and baseline claims is obfuscated by the lack of freely available, standardized, georeferenced project boundaries^14^. Carbon credits are managed by registries, typically non-profits, that have broad discretion over data sharing. This has resulted in many project boundaries not being available for independent emissions verification^12,17^. Moreover, project boundaries are spread across various registries and lack standardization in their submission requirements. Many project boundaries exist only as images and maps within project documentation, rather than as independent geospatial data. These challenges make finding, consolidating, and utilizing project boundaries onerous, and are primary reasons why existing analyses of nature-based carbon projects have utilized relatively small project databases compared to the total (>600) number of existing NBS projects^1,12,17^. We believe it is important the carbon offset boundaries be made publicly available. In response to criticism of carbon projects, the Integrity Council for the Voluntary Carbon Market (ICVCM) was formed as a multi-stakeholder-led independent governance body designed to improve standards within the voluntary carbon market. One of the ICVCM’s Core Principles is transparency, and many protocols under which projects are developed mandate that boundary information is shared. For independent auditors to evaluate carbon projects in a comprehensive manner, spatial boundary data are required.

To ameliorate challenges associated with NBS project boundaries, we have compiled a global database of spatial boundaries from 575 nature-based forest carbon projects across 55 countries. Three project types are represented in the database: avoided deforestation (AD), improved forest management (IFM), and afforestation, reforestation, and re-vegetation (ARR). The projects in the database are listed on six carbon registries: American Carbon Registry, BioCarbon Standard, Climate Action Reserve, EcoRegistry, Gold Standard, and Verra. Boundary data were obtained from carbon registries, direct communication with project managers, and manual georeferencing. We present the systematic framework used for the compilation of this open-access database.

This effort seeks to democratizes access to project boundaries for future measurement, reporting and verification (MRV) initiatives. Additionally, this database will facilitate more comprehensive analyses of the climate benefits provided by NBS. Lastly, the development of the database aligns with the objectives outlined by regulatory and industry standards bodies, ICVCM’s Core Carbon Principles, which mandate transparency in carbon project reporting.

## Methods

### Project boundary aggregation

**Fig. 1:**
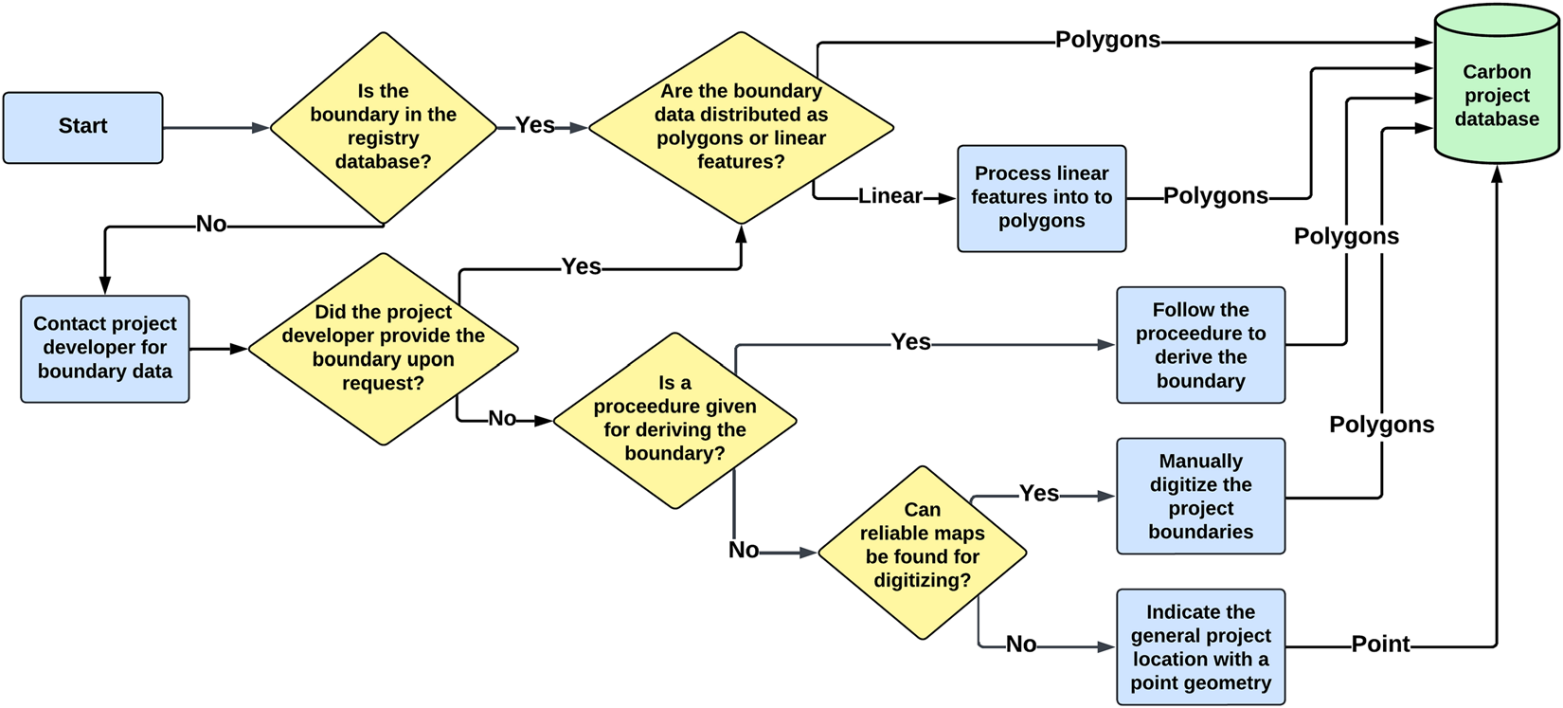
A flowchart depicting the procedures followed when aggregating the boundary data for the carbon project database.

**Fig. 2:**
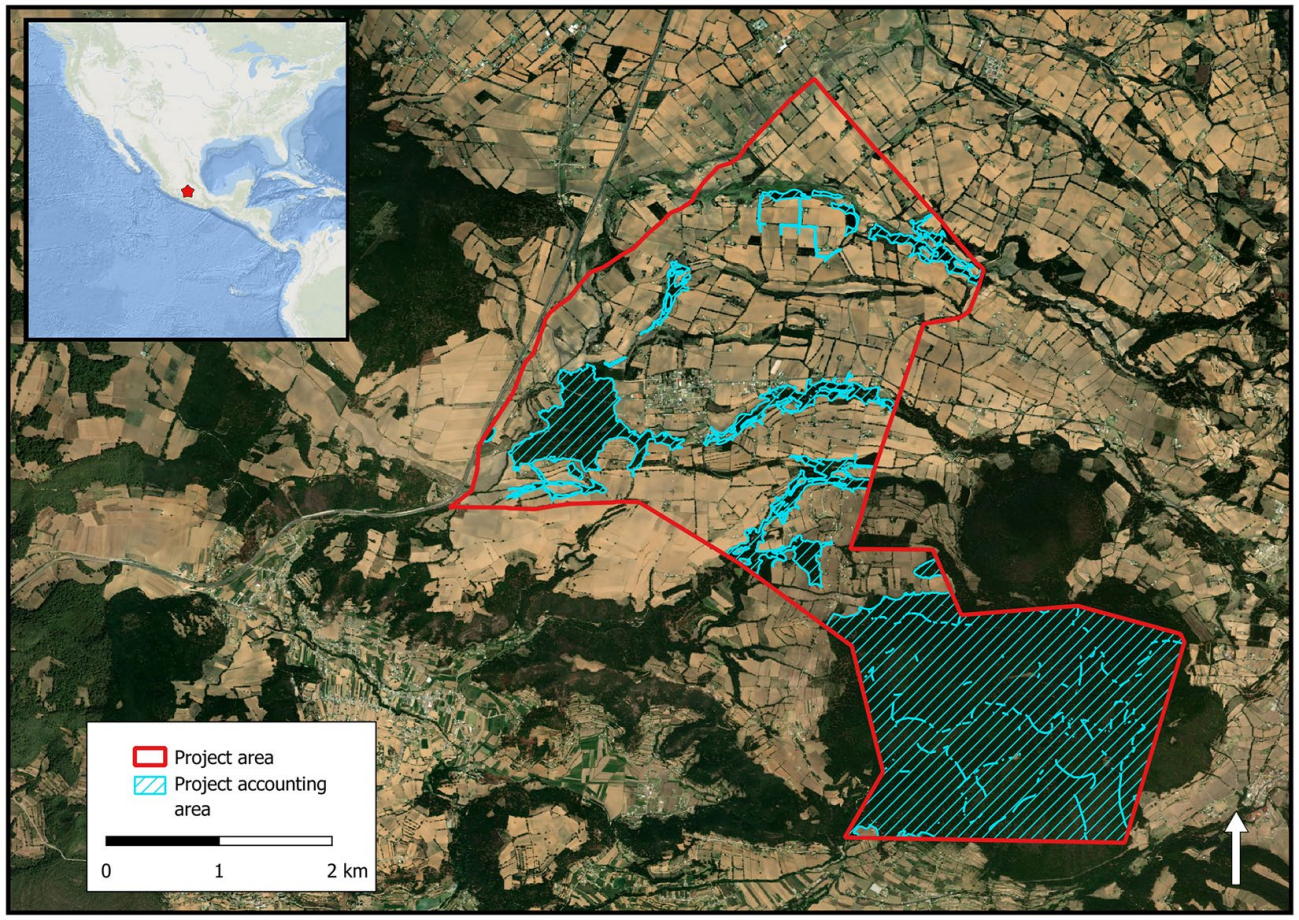
In the database, we distinguish between the project area and the project accounting area. The project area and the project accounting area for the forest carbon capture in the Ejido El Potrero (CAR1454) project registered under the Climate Action Reserve are depicted.

Project data were compiled via a combination of web-scraping, personal communication, and manual georeferencing (Fig. 1). A complete list of all projects registered under six registries was compiled (Table 1). For each project, all publicly available registry data including project description documents (PDD), monitoring reports, verification reports, and project boundary geospatial files were accessed. If project boundaries were made available, direct integration into the database was done following metadata standardization. We distinguish between project area and project accounting area (Fig. 2) to facilitate verification of project claims regarding carbon sequestration calculations. The project area is defined as the geographical area where the project participants implement activities to reduce deforestation. The project accounting area is defined as the geographical area of the project that was used to calculate carbon credit issuance. In 460 (83%) instances, the project accounting area and the project area are the same.

**Table 1:** A summary of the registry databases that were searched when aggregating the dataset.

Registry	Access date	Date of oldest project	Link to Registry
American Carbon Registry	01/20/2024	05/14/1999	^26^
BioCarbon Standard	09/07/2023	01/01/2010	^27^
Climate Action Reserve	03/14/2024	11/14/2001	^28^
EcoRegistry	07/25/2023	03/01/2007	^29^
Gold Standard	09/06/2023	01/01/1995	^30^
Verra	10/25/2023	04/05/1999	^31^

The date of database access, the date of the oldest project registered within each database, and the registry access links are provided. Document types retrieved from all registries were project description documents (.pdf, .docx) and project spatial boundaries (kml, .kmz, .shp).

In the event that geospatial data were not made available, points of contact for projects were contacted via email addresses listed in the PDD. Standardized emails requesting permission to include project’s boundary within the database were sent. If no initial response was received, a reminder email was sent within a two week window. If no reply was received to initial or follow-up email, manual georeferencing and digitizing of the project boundary was performed.

### Manual georeferencing & digitization

Project boundaries were manually georeferenced using QGIS (v3.32.2)^18^. First, the map with the clearest boundary within the PDD was identified and overlaid on OpenStreetMap^19^ and Bing Satellite Imagery^20^ basemaps. Each map was then georeferenced with the basemap layers using at least 4 ground control points. The geometries depicted on the georeferenced map were then manually digitized. Digitized geometries were then consolidated into a single geospatial layer representing the project area or project accounting area.

For nine projects, the project boundaries were distributed by registries as a set of linear features rather than polygons. If this was the case, linear geometries were processed to obtain the boundary data. To do so, the project’s linear features first were buffered by 30 m. Holes in the resulting geometry were then eliminated using the “Delete Holes” tool in QGIS. An inverse (i.e., negative) buffer of 29m was then applied to obtain the final polygon geometry. All geospatial boundaries were standardized to use geographic coordinates and the World Geodetic System 1984 datum (EPSG:4326).

For six projects, the project boundaries were not available in the registry but a detailed description about how the project boundaries were created was outlined in the PDD. If this was the case, we followed the steps mentioned in the PDD to recreate either the project area, the project accounting area, or both. The Mai Ndombe (VCS934) project area was downloaded from the registry. Then, the project accounting area was derived by removing portions of the project area that were within a 2.5km diameter buffer placed around each community within the concessions for the purposes of planned forest activities. The Lower Zambezi (VCS1202) project area was downloaded from the registry. Then, the project accounting area was obtained by removing all areas with a slope greater than 20 degrees using the ASTER Global Digital Elevation Model (GDEM)^21^. The 20 degree threshold was prescribed by the developer in the PDD. The Suruí Forest (VCS1118) reference area boundary was available in the registry. A land use land cover (LULC) map of the project reference area was obtained using the Copernicus Global Land Cover (CGLC) dataset^22^. The deforested areas in the LULC layer were extracted and a 1km buffer was created around these locations to obtain the project area. The Chocó-Darién Conservation Corridor (VCS856) project accounting area was derived by excluding portions of the project area (downloaded from the registry) that were steeper than 33 degrees, as described in the PPD, using the ASTER GDEM to derive the project accounting area. The Magnolios de Yarumal (VCS2317) project area was downloaded from the registry. The project accounting area was then created by excluding areas that were not forested prior to 2016 using the CGLC LULC dataset. The Serra do Amolar (VCS2566) project area boundary was downloaded from the registry. The project accounting area was created by removing areas that were not forested prior to 2016 using the CGLC LULC dataset from the project area.

Lastly, for 42 projects, we were unable to derive a geospatial boundary due to insufficient information being available. For these projects, we were able to identify the general location of the project and related information such as the project type. In these cases, the project was entered into the database as a point geometry.

### Database compilation results

**Fig. 3:**
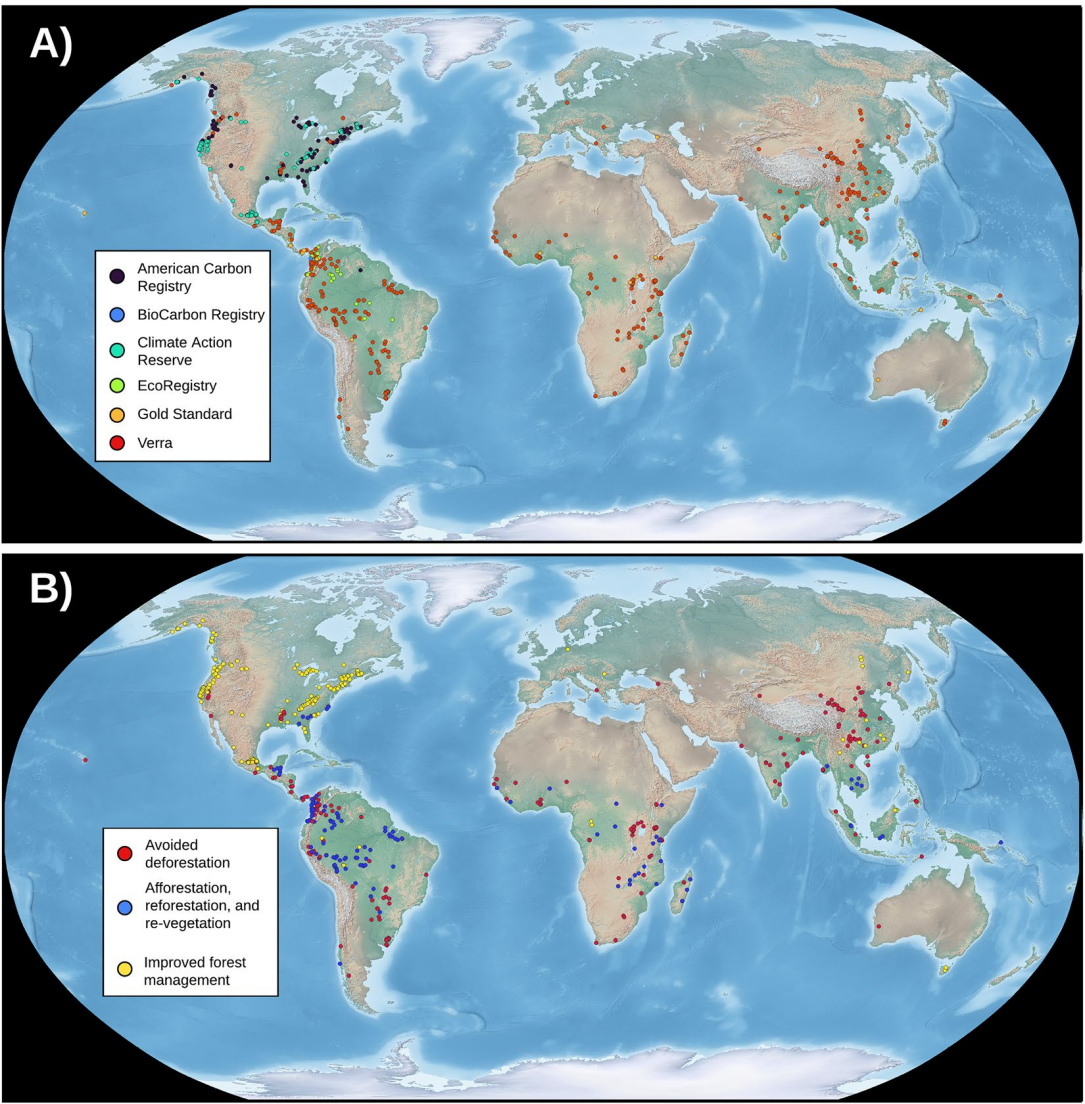
The location of carbon offset projects contained within the database. The registry under which the projects were enrolled (**A**) and the project type (**B**) are depicted. The data are displayed over the Natural Earth basemap dataset^32^.

**Fig. 4:**
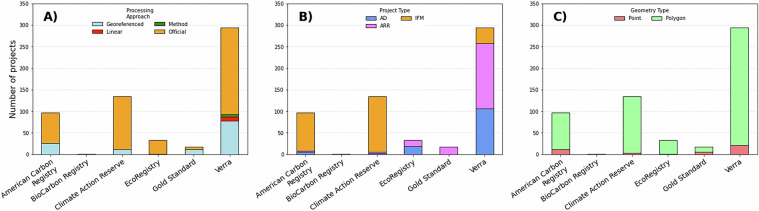
A summary of the contents of the carbon project database. The methodology use to obtain the project boundary (i.e., official boundaries, georeferencing and digitizing, processing linear geometries, or following developer describe procedure) (**A**), the distribution of project types (**B**), and the proportion of polygon vs. point geometries acquired for each registry are depicted. AD, avoided deforestation; IFM, improved forest management; ARR, afforestation, reforestation, and re-vegetation.

A total of 575 geospatial boundaries from 55 countries were compiled from six carbon registries (Fig. 3). The database contains information on 252 IFM, 190 ARR, and 133 AD projects (Fig. 4). The core components of the database consist of registry provided project boundaries (*n* = 433, 75.3%) and manually georeferenced and digitized boundaries (*n* = 127, 22.1%). Nine project locations (~1.5%) were distributed as linear features that were processed into polygon boundaries, and six project boundaries (~1.5%) were created using a developer given protocol. Verra projects were mostly either AD (*n* = 106) or ARR (*n* = 152). The majority of IFM projects were registered under the Climate Action Reserve (*n* = 128) or American Carbon Registry (*n* = 88) (Table 2). The majority of IFM projects were located in the United States (*n* = 171), Mexico (*n* = 52), and China (*n* = 11). The database contains 533 polygon boundaries (92.7%) and 42 point locations (7.29%) where only the general project location could be determined. The countries with the greatest number of projects are the United States of America (*n* = 189), Colombia (*n* = 58), Mexico (*n* = 55), China (*n* = 48) and Brazil (*n* = 41) (Table 3).

**Table 2:** Summary of project types and digitization metrics across six carbon registries in the global database.

Registry Name	Project types			Total projects	% Digitized	% Polygon
	Num. IFM	Num. AD	Num. ARR			
American Carbon Registry	88	5	3	96	27.1	88.5
BioCarbon Registry	0	1	0	1	0.0	100.0
Climate Action Reserve	128	2	4	134	8.2	97.8
EcoRegistry	0	19	14	33	3.0	97.0

Registry Name	Project types			Total projects	% Georeferenced	% Polygon
	Num. IFM	Num. AD	Num. ARR			
Gold Standard	0	0	17	17	64.7	64.7
Verra	36	106	152	294	26.5	92.9
**Totals**	**252**	**133**	**190**	**575**	**22.1**	**92.7**

It includes the percentage of georeferenced projects (excluding linear geometries following developer protocols) and the percentage of complete polygon boundaries (excluding point geometries).

**Table 3:** Country-wise project data including number of IFM, AD, ARR projects, total projects, percentage georeferenced, and percentage polygon.

Country	Num. IFM	Num. AD	Num. ARR	Total projects	% Georeferenced	% Polygon
Albania	0	0	1	1	0.0	100.0
Argentina	0	0	1	1	0.0	100.0
Australia	3	0	1	4	50.0	100.0
Belize	0	4	0	4	0.0	100.0
Benin	0	1	0	1	0.0	100.0
Bolivia	1	3	1	5	60.0	80.0
Brazil	1	30	10	41	12.2	97.6
Cambodia	0	4	0	4	50.0	100.0
Cameroon	1	0	0	1	0.0	100.0
Canada	3	0	0	3	33.3	100.0
Central African Republic	0	1	0	1	100.0	100.0
Chile	0	1	2	3	66.7	100.0
China	11	0	37	48	18.8	79.2
Colombia	1	34	23	58	29.3	94.8
Costa Rica	0	0	1	1	100.0	0.0
Democratic Republic of the Congo	0	2	1	3	33.3	66.7
Ethiopia	0	1	2	3	66.7	100.0
Georgia	0	0	1	1	100.0	100.0
Germany	1	0	0	1	100.0	0.0
Ghana	0	0	5	5	0.0	100.0
Guatemala	0	3	3	6	33.3	100.0
Guinea-Bissau	0	1	0	1	100.0	100.0
India	0	0	11	11	0.0	100.0
Indonesia	0	4	2	6	16.7	100.0
Kenya	0	4	9	13	7.7	100.0
Laos	0	1	1	2	0.0	100.0
Madagascar	0	3	1	4	0.0	100.0
Malawi	0	1	1	2	50.0	50.0
Malaysia	2	0	0	2	0.0	100.0
Mali	0	0	1	1	0.0	100.0
Mexico	52	0	3	55	7.3	100.0
Mozambique	0	1	1	2	50.0	100.0
Myanmar	0	1	3	4	50.0	75.0
Nicaragua	0	0	3	3	0.0	100.0
Niger	0	0	1	1	0.0	100.0
Pakistan	0	0	1	1	0.0	100.0
Panama	0	1	3	4	50.0	50.0
Papua New Guinea	1	1	0	2	50.0	100.0
Paraguay	0	2	5	7	0.0	100.0
Peru	1	13	7	21	38.1	95.2
Philippines	0	0	2	2	0.0	100.0
Republic of the Congo	1	0	2	3	0.0	100.0
Romania	1	0	0	1	0.0	100.0
Russian Federation	1	0	0	1	100.0	0.0
Rwanda	0	0	1	1	100.0	0.0
Senegal	0	0	2	2	50.0	50.0
Sierra Leone	0	1	1	2	0.0	100.0
South Africa	0	0	4	4	75.0	100.0
Tanzania	0	3	2	5	0.0	100.0
Timor-Leste	0	0	1	1	100.0	0.0
Uganda	0	0	11	11	27.3	90.9
United States of America	171	7	11	189	21.7	92.6
Uruguay	0	0	10	10	20.0	100.0
Zambia	0	4	1	5	40.0	100.0
Zimbabwe	0	1	0	1	0.0	100.0

It includes the percentage of georeferenced projects (excluding linear geometries following developer protocols) and the percentage of complete polygon boundaries (excluding point geometries)

Project developers were contacted to request boundary data for 149 projects. Project developers declined to provide or did not respond to our request for 141 of the boundaries (94.6%). The Gold Standard project had the greatest proportion of project listed on their registry which lacked official boundary data (64.7%, *n* = 11). The American Carbon Registry lacked project boundaries for 27.1% (*n* = 26) of the projects. The Verra project registry lacked project boundaries for 26.5% (*n* = 93) of the projects. Proportionally, fewer project boundaries needed to be aggregated from the Climate Action Reserve registry (8.2%, *n* = 11) and from the EcoRegistry (3%, *n* = 1).

## Data Records

The global database of nature-based carbon offset project boundaries is hosted on Zenodo. The data are open source and made available at Zenodo^23^. New versions of the database will be released periodically as additional project boundaries are aggregated.

The database of project locations was distributed as a set of 6 continental GeoPackage files (.gpkg). The attributes associated with each entry in the database are described in Table 4. A comma-separated value (CSV) file, containing a database index, is included to facilitate locating individual projects within the geodatabase. The CSV file contains the same attributes as the geopackages with an additional field indicating which continent each project is located on. Additionally, the CSV indicates if the Project Area, Project Accounting Area, and Reference area are present using a binary value (1 for present, 0 for absent). By default, when the geopackages are loaded, the project area is defined as the geometry. We include a Python script that demonstrates how to process the geopackage files and specify the project accounting areas as the geometry.

**Table 4:** Each of the records in the GeoPackages that constitute the carbon project database contain the following attributes.

Data Field	Description
Project Name	The name of the carbon project as given in the documentation.
ProjectID	A combination of the registry abbreviation of the registry and project number
Registry Name	The name of the registry where project information is hosted. Possible values are: ‘American Carbon Registry’, ‘BioCarbon Registry’, ‘Climate Action Reserve’, ‘EcoRegistry’, ‘Gold Standard’, ‘Verra’
Methodology	The name of the methodology used for the project’s implementation.
Project Type	The type of forestry carbon offset program. One of the following: “ARR”, “AD”, or “IFM”
Country	The country where the project is located.
Project Developer Name	Name of the entity or individual organizing, proposing or advocating a particular carbon offset project. In case of multiple entities/individuals, only the first name is recorded here.
Project Start Date	Date of the start of the crediting period (mm/dd/yyyy).
Project End Date	Date of the end of the crediting period (mm/dd/yyyy).
Date of Entry	Date when the project information was added to the database (mm/dd/yyyy)
Processing Approach	One of four possible values: “Official”, “Georeferenced”, “Linear”, or “Method”. “Official” if the canonical boundary was obtained from a registry or the project developer. “Georeferenced” if the boundary data was obtained via georeferencing and digitizing maps obtained from the project documents. “Linear” if the boundary data were distributed as linear features that were processed into geometries. “Method” if a developer specified protocol was followed to obtain the boundary data.
PD Declined to Provide	One of: “Yes”, “No”, “N/A”. “Yes” if the project developer was contacted for the project geometry but declined to provide the necessary information. “No” if the project developer provided the geometries or sufficient information to create the project geometry. “N/A” if the geometry was available from a registry and the developer was not contacted.
Geometry Type	One of: “Point” or “Polygon”. Indicates if the geometry in the database is a point or a polygon.
Project Area	The well-known text (WKT) representation of the geographical area where the project participants implement activities to reduce deforestation.
Project Accounting Area	The WKT representation of the geographical area of the project which was used to calculate carbon credits. If the project area is the same as the project accounting area, then the project accounting area is not defined.
Project Reference Region	The well-known text (WKT) representation of the geographical area of the project from where historical and current deforestation and forest degradation quantities and trends are obtained. This is not always defined.
Comment	Notes about how manual referencing was carried out or other relevant information.

## Technical Validation

Manually georeferencing and digitizing project boundary data can be challenging due to cartographic properties of the maps in the PDD. The backgrounds of the maps in the PDDs frequently used stylized basemaps that feature fewer precise indicators of a particular location than satellite image basemaps. Similarly, the spatial extent, spatial scale, and aesthetic choices (e.g., boundary line thickness) impacted georeferencing. In many cases, PDD maps were better suited for visualizing the project in its geographic context as opposed to being optimized for georeferencing. Consequently, the difficulty of accurately georeferencing a given project can vary.

**Fig. 5:**
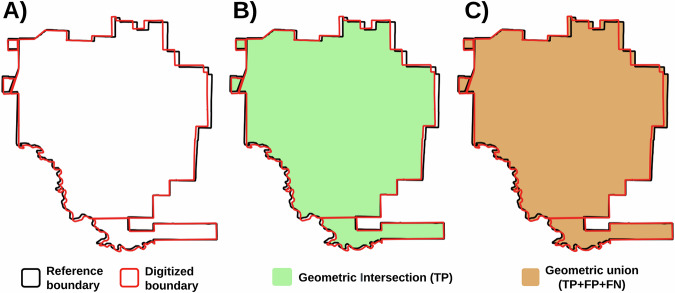
The reference polygon (black) and the manually digitized polygon (red) for the Cook’s Branch Conservancy project (ACR257). True positives (TP) corresponds to the intersection of the reference and digitized polygons. The union of the two boundaries encompasses the true positives, the false positives (FP), and the false negatives (FN).

We conducted an accuracy assessment to quantify the reliability of the georeferencing and digitization procedures. This involved comparing reference polygons, project boundaries that were provided by the registry, with manually digitized polygons that were created using maps found in PDD. We randomly selected 10 projects from the database to assess digitizing accuracy (Table 5). We report the digitizing accuracy using three metrics, Intersection over Union (IoU), precision (i.e., user’s accuracy), and recall (i.e., producer’s accuracy)^24,25^. These are formulated as:

1 $$IoU=\frac{TP}{TP+FP+FN}$$

2 $$Precision=\frac{TP}{TP+FP}$$

3 $$Recall=\frac{TP}{TP+FN}$$

where TP (true positives) represent locations were the reference and digitized polygons intersect, FP (false positives) represent areas that were digitized that do not exist in the original reference data, and FN (false negatives) represent areas in the reference dataset that were not included in the digitized polygons (Fig. 5).

**Table 5:** The ten projects, for which official boundaries were available, that were randomly selected from the database to assess the accuracy of our georeferencing and digitizing procedure.

Project Name	Project ID	Project Type	Area (km^2^)	IoU	Precision	Recall
Baka Rokarire	ER53	AD	7128.0	0.983	0.992	0.992
Buck Mountain	CAR1066	IFM	50.6	0.946	0.973	0.972
Ganados Bosques	ER30	ARR	3.0	0.911	0.946	0.961
Hawk Mountain	ACR375	IFM	9.7	0.960	0.978	0.981
Juina	ER139	AD	2883.0	0.992	0.997	0.995
Keo Seima	VCS1650	AD	1879.3	0.991	0.997	0.994
Lubrect	ER1332	IFM	82.0	0.950	0.976	0.974
Pacajai	VCS981	AD	1231.6	0.973	0.984	0.989
Santa Ines	ER97	ARR	2.9	0.952	0.973	0.978
Sustainable Mountain	CAR1067	IFM	8.6	0.970	0.984	0.986

Our assessment indicated that georeferencing was performed with a high degree of accuracy. The IoU score of 0.96 ( ± 0.03) indicates a high degree of agreement between the reference and digitized boundary data. The precision (0.98  ± 0.02), and recall (0.98  ± 0.01) were almost equal indicating a low and balanced error rate between FP and FN values. Digitizing accuracy was observed to often be higher on larger polygons than smaller polygons. The IoU score of the 5 largest projects assessed was 0.98 ( ± 0.02) while IoU score of the 5 smallest projects was 0.9478 ( ± 0.02). Intuitively, this makes sense as larger, contiguous projects the TP term will dominate the FP and FN terms. However, it is noted that this will likely not be the case for projects whose total area is large but that are subdivided into many small areas.

## Usage Notes

The presented carbon project database is designed to facilitate MRV initiatives. Analysts do however need to consider some of the database’s limitations. This database does not represent a census of nature-based carbon projects and does not contain all varieties of nature-based carbon projects. Users should verify that any georeferencing inaccuracies will not significantly impact their analyses. The boundaries included in the database reflect the data available in the registries at the time of access, with some projects regularly updating their information. Users must carefully distinguish between overall project areas and project accounting areas based on their specific analysis requirements. For example, questions that assess project crediting should use the project accounting areas. Lastly, we note that we were unable to assess the accuracy of the boundaries constructed from linear features or from a developer provided protocol. This is because we lack the original boundaries to make a comparison. Users of the database are encouraged to evaluate these project boundaries on a case-by-case basis.

## Data Availability

Project boundary data were derived using QGIS. No code, other than the demonstration Python script provided in the data repository, are available.
