# Author Correction: An open-access database of nature-based carbon offset project boundaries

**DOI:** 10.1038/s41597-025-05005-9

**Published:** 2025-04-22

**Authors:** Akshata Karnik, John B. Kilbride, Tristan R. H. Goodbody, Rachael Ross, Elias Ayrey

**Affiliations:** 1 Renoster Systems Inc., 21750 Hardy Oak Blvd Ste 104, PMB 37519, San Antonio, TX 78258-4946 United States of America; 2 Carbon Direct, Vancouver, British Colombia, Canada; 3 Carbon Direct, New York, New York, United States of America

**Keywords:** Forestry, Environmental impact, Forestry, Environmental impact

Correction to: *Scientific Data* 10.1038/s41597-025-04868-2, published online 5 April 2025

In this article the author’s name John B. Kilbride was incorrectly written as Jack B. Kilbride. The original article has been corrected.

